# Dissection of a Double Monster like the Siamese Twins

**Published:** 1874-02-15

**Authors:** 


### DISSECTION OF A DOUBLE MONSTER LIKE THE SIAMESE TWINS.

THE recent death of the Siamese Twins, Eng and Chang, in North Carolina, recalls attention to this famous monster. These persons were born in Siam, and brought to this country, while children, for exhibition. They were connected by a band attached to the mesial line, and extending from the umbilicus to about the lower end of the sternum. By their continued efforts to avoid the inconvenient face-to-face position, they had gradually stretched the connecting tissue into a band long enough to allow them to stand side by side.

The newspaper account of their death is, that one of them was attacked by pneumonia, which proved fatal, and that the other, after great alarm and agitation, died two hours and a half later, of causes not clearly definable. The statement is also made that no *post-mortem* examination was allowed, but that the bodies were packed in charcoal, where, by last accounts, they were rapidly decomposing.

Some years ago, Prof. E. Andrews, at that time Professor of comparative, and demonstrator of human, anatomy, in the University of Michigan, received, from a physician of that State, a double monster, almost exactly like the Siamese Twins, which had recently been delivered under his care. The woman presented at the *os uteri* the cephalic extremities of two children, which seemed to be attached to each other in some mysterious way, so that the physician was unable to separate them enough to allow them to come down one at a time. After he had used his best endeavors in vain, by introducing his hand, the woman, by some tremendous uterine contractions, expelled them both together. The cord was still pulsating, but no respiration occurred, and the monster soon died. On examination of the uterus for the placenta, a third child was found present, which was dead when delivered.

Prof. Andrews carefully examined the bodies, so far as the connected parts were concerned. The attachment was, apparently, precisely like that of the Siamese Twins, commencing at the common umbilicus, and extending upward to near the point of sternum. On opening the parts, the livers were found to be firmly attached to each other, so that it might be correcly said, that there was one common double liver extending across from one body to the other. Below the connecting mass of hepatic tissue, the two abdominal cavities were separated by a thin peritoneal septum, which, however, had an oval opening of considerable size through it, so that the two cavities communicated freely with each other. The stomach and intestines had no connection ; and the hearts and lungs were, in like manner, entirely separate.

If the Siamese Twins were united like this pair, it is evident that any attempt to separate them must have proved fatal, a conclusion which accords with that of the European surgeons, who investigated the case of Chang and Eng.

The specimen dissected by Prof. Andrews was placed in the museum of the Medical Department of the University of Michigan, where it may still be seen, unless it has since been removed.

